# Development and Characterization of Collagen–Methylcellulose Sponge-like Matrices for Indomethacin Release in Wound Dressing Applications

**DOI:** 10.3390/ph19060918

**Published:** 2026-06-10

**Authors:** Maria-Teodora Pițuru, Mădălina Georgiana Albu Kaya, Denisa Ioana Udeanu, Cristina Elena Dinu-Pîrvu, Elena-Emilia Tudoroiu, Ioana Luca, Lăcrămioara Popa, Valentina Anuța, Zina Vuluga, Bruno Ștefan Velescu, George Mihail Teodorescu, Elena Denisa Trandafir, Mihaela Violeta Ghica

**Affiliations:** 1 Department of Animal Productions and Public Health, Faculty of Veterinary Medicine, University of Agronomic Sciences and Veterinary Medicine, 105 Splaiul Independenței Blvd., 050097 Bucharest, Romania; maria.pituru@fmvb.usamv.ro; 2 Biobase, Faculty of Pharmacy, “Carol Davila” University of Medicine and Pharmacy, 6 Traian Vuia Str., 020956 Bucharest, Romania; 3 Department of Collagen, Division of Leather and Footwear Research Institute, National Research and Development Institute for Textiles and Leather, 93 Ion Minulescu Str., 031215 Bucharest, Romania; 4 Department of Clinical Laboratory and Food Safety, Faculty of Pharmacy, “Carol Davila” University of Medicine and Pharmacy, 6 Traian Vuia Str., 020956 Bucharest, Romania; 5 Department of Physical and Colloidal Chemistry, Faculty of Pharmacy, “Carol Davila” University of Medicine and Pharmacy, 6 Traian Vuia Str., 020956 Bucharest, Romania; cristina.dinu@umfcd.ro (C.E.D.-P.); ioana.luca@drd.umfcd.ro (I.L.); lacramioara.popa@umfcd.ro (L.P.); valentina.anuta@umfcd.ro (V.A.); trandafirdenisa@yahoo.com (E.D.T.); mihaela.ghica@umfcd.ro (M.V.G.); 6 Innovative Therapeutic Structures Research and Development Center (InnoTher), “Carol Davila” University of Medicine and Pharmacy, 6 Traian Vuia Str., 020956 Bucharest, Romania; 7 National Institute of Forensic Medicine “Mina Minovici”, 042122 Bucharest, Romania; emiliatudoroiu@yahoo.com; 8 National Institute for Research & Development in Chemistry and Petrochemistry–ICECHIM, 202 Spl. Independenței, 060021 Bucharest, Romania; zvuluga@icechim.ro (Z.V.); george.teodorescu@icechim.ro (G.M.T.); 9 Department of Pharmacology and Clinical Pharmacy, Faculty of Pharmacy, “Carol Davila” University of Medicine and Pharmacy, 6 Traian Vuia Str., 020956 Bucharest, Romania; bruno.velescu@umfcd.ro

**Keywords:** collagen, methylcellulose, indomethacin, wound healing, sponge-like matrices

## Abstract

**Background**: Interest in advanced wound dressings for clinical applications is increasing, with biopolymer-based formulations emerging as an effective strategy for wound management. **Objectives**: This study aimed to develop and characterize sponge-like biopolymeric matrices for the topical delivery of indomethacin as a model anti-inflammatory drug. **Methods**: Matrices were prepared by combining collagen and methylcellulose (MC) gels in varying ratios, followed by lyophilization. Physicochemical characterization included FT-IR, SEM, contact angle, and water absorption analysis. Biological evaluation involved enzymatic degradation, while biopharmaceutical and pharmacological assessments included in vitro drug release and in vivo testing in Wistar rats with experimentally induced burns. **Results**: FT-IR analysis confirmed that collagen’s triple-helical structure was preserved in the presence of MC and indomethacin for the samples with maximum 25% methylcellulose. SEM analysis revealed a microporous network with integrated cellulose fibers, where pore size decreased with higher MC content. Compressive strength measurements demonstrated enhanced mechanical resistance with increasing MC content, indicating improved structural stability of the matrices. Moreover, increased MC content led to higher contact angle values but maintained hydrophilicity, while formulations with up to 25% MC exhibited good absorption capacity and structural integrity. Enzymatic degradation studies confirmed that matrices with at least 75% collagen content maintained their structural integrity over time, favoring prolonged application and sustained drug delivery. In vitro drug release followed a biphasic profile, supporting rapid initial anti-inflammatory action followed by gradual release of the drug. In vivo animal studies demonstrated accelerated wound healing in treated rats for all tested matrices. **Conclusions**: Overall, the developed indomethacin-loaded biopolymeric matrices showed promising structural, functional, and therapeutic properties for effective wound treatment.

## 1. Introduction

Acute skin injuries, particularly burns, are a major public health problem worldwide, associated with significant morbidity and mortality [1]. According to recent epidemiological data, in 2021, over 249 million cases of burns were reported annually worldwide, a significant proportion of which required hospitalization and specialized medical care. Until 2025, there is a projection of an increase of 6.42%, especially in Eastern Europe and Latin America [2]. International health organizations estimate that severe burns are responsible for hundreds of thousands of deaths annually (over 180,000), disproportionately affecting populations in low- and middle-income countries [3]. In addition, the socio-economic impact of these injuries is considerable, including high treatment and recovery costs, as well as impaired quality of life for patients [4].

The healing process of skin lesions is a complex one, involving a sequence of well-defined stages: hemostasis, inflammation, proliferation and tissue remodeling [5]. From these, the inflammatory phase plays an essential role in initiating the repair process, but excessive or prolonged inflammation can have negative effects, leading to delayed healing and the formation of pathological scars [6]. In the case of burns, the inflammatory response is often intensified, requiring an appropriate therapeutic intervention to limit the adverse effects of the inflammation and to promote tissue regeneration [7].

Thus, multifunctional modern dressings (hydrogels, aerogels, foams, sponges, films, nanofibers) have evolved significantly compared to traditional ones, being designed not only as physical protective barriers, but also as active systems due to their ability to deliver the loaded drug in a controlled manner, supporting and accelerating the healing process [8,9]. Advanced dressings are designed to maintain an optimal moist environment at the lesion level, to allow gas exchange, to absorb exudate and, at the same time, to prevent microbial contamination [10]. Moreover, the integration of bioactive agents into the structure of these dressings represents an innovative direction, which allows direct intervention on the physiological mechanisms involved in healing [11].

There are many polymers that vary according to their chemical nature: natural (collagen, gelatin, cellulose, keratin, chitosan), semisynthetic (sodium alginate and cellulose derivatives), and synthetic (polyvinyl alcohol, polyvinylpyrrolidone, polyethylene glycol, polyurethan), which exhibit versatile properties, making them promising candidates for the development of new, modern dressings [12,13].

Collagen-based materials have attracted great interest in the field of tissue engineering and the development of wound dressings. Collagen is the main structural protein of the extracellular matrix and plays a key role in supporting tissue architecture and facilitating cellular interactions [14]. Due to its biocompatibility, biodegradability and regeneration-promoting properties, collagen provides an ideal support for the development of advanced matrices used in the treatment of various skin lesions [15]. However, the use of collagen in its pure form can be limited by poor mechanical properties, which is why it is frequently combined with other polymers [16].

Methylcellulose (MC), a semisynthetic derivative of cellulose, is a hydrophilic polymer widely used in pharmaceutical applications due to its ability to form gels and retain water. The outstanding mechanical and chemical properties of MC make it a remarkable polymeric material to be used for multiple medical applications, alone or in combination with other polymers [12]. In our previous review [17], cellulose derivatives were systematically analyzed for wound healing applications, highlighting that while sodium carboxymethylcellulose is extensively studied, other derivatives such as methylcellulose and hydroxyethylcellulose (HEC) remain comparatively less explored despite their promising characteristics.

Building on these findings, we subsequently developed collagen-based spongious matrices incorporating cellulose derivatives, including MC and HEC, and demonstrated their favorable physicochemical properties and biocompatibility with human adult dermal fibroblasts. The combination of collagen with methylcellulose resulted in porous spongious matrices with improved characteristics, including enhanced mechanical stability, high exudate absorption capacity, and good biocompatibility. These characteristics are essential for maintaining a moist environment conducive to healing and ensuring adequate contact between the dressing and the wound surface. Moreover, these scaffolds represent suitable supports for incorporating various drugs and developing drug delivery systems for medical use [18].

Based on these considerations, the present study focuses on the development of collagen–methylcellulose systems to form stable matrices suitable for wound dressing applications.

In this context, an important direction in the development of modern dressings is the incorporation of active substances with a therapeutic role, such as anti-inflammatory agents, local anesthetics, antimicrobials or growth factors [19]. Non-steroidal anti-inflammatory drugs (NSAIDs) are frequently used in the management of inflammation and pain associated with skin lesions [20].

Indomethacin, a non-selective non-steroidal anti-inflammatory drug (NSAID), was selected as a model anti-inflammatory agent due to its well-established pharmacological activity in reducing inflammation and pain, which are critical factors during the early phase (24–48 h) of the wound healing process, particularly in burn injuries [21]. It acts by inhibiting cyclooxygenases, enzymes involved in the synthesis of prostaglandins, key mediators of inflammation [22].

Systemic administration of NSAIDs, especially orally, is, however, associated with several adverse effects, including gastrointestinal irritation and ulceration, renal damage and an increased risk of cardiovascular events [23]. These limitations have led to the exploration of alternative routes of administration, among which topical administration presents multiple advantages [24]. Local application of NSAIDs allows for the achievement of adequate therapeutic concentrations directly at the lesion site, while reducing systemic exposure and the risk of adverse reactions. In addition, topical administration facilitates a controlled release of active substance, which may contribute to the maintenance of a sustained therapeutic effect [25]. Indomethacin has been widely investigated in topical and localized delivery systems due to its suitable physicochemical properties, which allow its incorporation into polymeric matrices and facilitate controlled release at the site of application [26].

Previous studies have successfully formulated indomethacin in various delivery platforms, including gel [27], nanoemulsion [28], films [29,30], sponge [31], and nanofibers [22], demonstrating its applicability for localized therapy. Moreover, when administered topically, indomethacin exhibits reduced systemic exposure, thereby minimizing potential gastrointestinal and cardiovascular side effects commonly associated with oral NSAID administration.

Based on these considerations, indomethacin was selected as a suitable model drug for incorporation into the developed collagen–methylcellulose-based matrices, enabling the evaluation of their potential as localized drug delivery systems for wound healing applications.

The composition ranges used for the collagen–methylcellulose matrices were selected based on our previous study. The results demonstrated that collagen and methylcellulose were biocompatible and preserved the native triple-helix structure of collagen at a maximum MC gel concentration of 30%. In addition, the developed scaffolds exhibited favorable swelling behavior and wettability, ensuring efficient absorption of wound exudates [18]. Based on these findings, the collagen: methylcellulose ratios selected in the present study were considered appropriate to achieve a balance between structural stability, hydration capacity, and biological performance required for wound dressing applications.

In the context of blending methylcellulose with various polymers, Puttawibul et al. [32] developed an in situ hydrogel using type I collagen derived from shark skin combined with MC, aimed at central nervous system regeneration. Structural and morphological analyses revealed that the hydrogels possess a complex architecture characterized by distinct collagen fibril networks, formed through molecular interactions between both polymers. An injectable formulation containing 2.5% MC and 0.1% collagen was designed to support cell encapsulation, demonstrating high mechanical strength and enhanced biocompatibility in cell culture studies [33]. Coban et al. [12] showed that methylcellulose–okra mucilage–Hypericum perforatum oil–gentamicin composite films exhibit a more porous structure and increased water vapor permeability after incorporation of bioactive compounds. They demonstrated antioxidant and antibacterial activity, as well as good biocompatibility, contributing to the acceleration of the healing process in vitro. Abaza et al. [34] showed that incorporation of curcumin-loaded zein–methylcellulose nanoparticles into chitosan films significantly improved their mechanical, antibacterial, and antioxidant properties. The developed systems demonstrated effective antibacterial activity, as well as high biocompatibility. In in vivo studies, these dressings significantly accelerated wound healing, as evidenced by reduced inflammation, increased re-epithelialization, and collagen deposition. Dixit et al. [35] showed that thermoresponsive injectable hydrogels based on keratin and methylcellulose exhibit high biocompatibility, antioxidant properties and antibacterial activity, favoring the proliferation and migration of fibroblasts. In vivo studies demonstrated a significant acceleration of full-thickness wound healing, with rapid re-epithelialization, enhanced angiogenesis and increased collagen deposition. In addition, the hydrogels led to almost complete wound closure and regeneration of skin structures, with minimal scarring, highlighting their potential as advanced dressings for the treatment of chronic skin lesions.

Therefore, the development of collagen and methylcellulose-based sponge-like matrices loaded with indomethacin represents an innovative approach in the field of advanced dressings for the treatment of acute skin injuries. These systems combine the favorable properties of biomaterials with the benefits of NSAID topical therapy, offering a promising solution for effective burn management [36].

## 2. Results and Discussions

### 2.1. Fourier Transform Infrared (FT-IR) Spectroscopy

**Figure 1:**
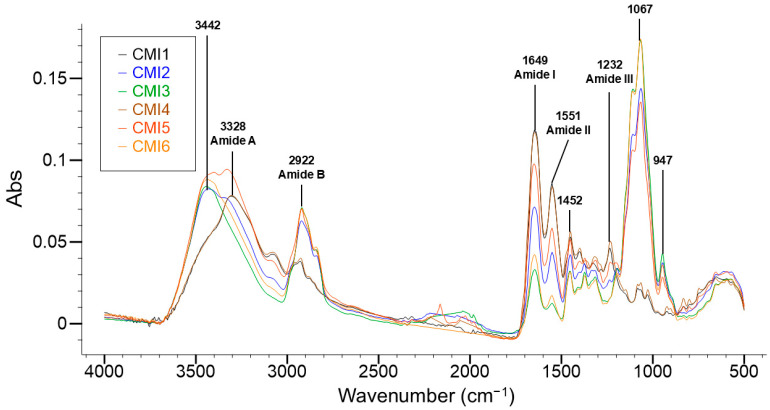
Overlapping FT-IR spectra of CMI1–CMI6 samples.

Fourier transform infrared (FT-IR) spectroscopy is an important technique for the characterization of polymeric blends used in wound healing applications [37]. In systems combining polymers such as methylcellulose with structural proteins like collagen, FT-IR analysis provides valuable information about their chemical composition, functional groups and the interactions occurring between the components [37,38]. In particular, FT-IR analysis is commonly used for the investigation of the protein secondary structure through the analysis of characteristic amide bands [39]. Among these, amide I, amide II, and amide III characteristic absorption bands are especially sensitive to the conformation of the polypeptide backbone and allow the evaluation of structural changes in collagen during material preparation and processing [40]. Preserving the native secondary structure of collagen, which is associated with its triple-helical organization, is crucial because it directly influences the protein’s mechanical stability, biocompatibility and biological activity [41,42]. Maintaining this structural integrity within methylcellulose–collagen blends is important for ensuring favorable physicochemical properties, such as structural stability, as well as biological functions including cell adhesion and tissue regeneration [43,44]. Therefore, FT-IR spectroscopy represents a key tool for studying the interactions between blend components and the preservation of the collagen structure in composite wound dressing materials. The FT-IR spectra of CMI1–CMI6 samples were analyzed to evaluate the chemical composition of the obtained polymeric matrices and to assess possible structural changes induced by the incorporation of indomethacin in different concentrations or by blending the two polymers. The FT-IR spectra obtained for CMI1–CMI6 samples are presented in Figure 1.

The obtained spectra show the typical absorption bands of both collagen and methylcellulose. Samples with 100% collagen gel (CMI1 and CMI4) and those with 75% collagen gel and 25% methylcellulose gel (CMI2 and CMI5) showed very similar spectral characteristics. These four samples exhibited specific bands of collagen. These include the amide I band around 1650 cm^−1^, corresponding mainly to C=O stretching vibrations of the peptide bond; the amide II band around 1550 cm^−1^ related to N-H bending and C-N stretching; and the amide III band around 1230 cm^−1^, which is generally associated with the vibrations of the polypeptide backbone [45,46,47]. Moreover, other important bands are those at 1450 cm^−1^ (attributed to the aliphatic radicals from the amino acids of the collagen molecule) [48], at 3300–3350 cm^−1^ (amide A, linked to N-H stretching), and at 2900 cm^−1^ (amide B that is due to the symmetric stretching of methylene groups) [49]. The preservation of these characteristic amide bands in CMI1, CMI2, CMI4, and CMI5 formulations indicates that the secondary structure of collagen remains intact after processing and after blending collagen gel with methylcellulose gel and indomethacin. For CMI3 and CMI6 matrices, the spectra present only the bands for amide B, I, II, and the bands at 1450 cm^−1^, while the bands for amide A and amide III cannot be identified, results that are in line with our previous work [18]. The apparent attenuation of the amide A and amide III bands observed in CMI3 and CMI6 matrices is likely associated with spectral overlap with the broad O-H stretching vibrations of methylcellulose, as well as band broadening effects commonly encountered in composite polymer systems. In contrast, the amide I and amide II bands remain clearly detectable, suggesting that the collagen backbone is largely maintained. Nevertheless, variations in hydrogen-bonding interactions and polymer–polymer organization may contribute to modifications in the overall network architecture. These molecular-level changes may be related to the more compact morphology further observed by SEM at higher methylcellulose content (50%), indicating a densification and rearrangement of the composite structure. Such microstructural organization may influence material performance, particularly mechanical behavior and biological response, by affecting mass transport and cellular interactions within the matrix.

For samples containing both collagen and methylcellulose (CMI2, CMI3, CMI5 and CMI6), additional characteristic bands specific to the cellulose derivative can be observed. A strong absorption band appears in the region around 1100–1050 cm^−1^, which corresponds to C-O-C stretching vibrations of ether linkages and glycosidic bonds in the polysaccharide backbone. This band is typical for cellulose derivatives (ethers) and confirms the presence of methylcellulose in the composite matrices. Other representative methylcellulose bands include the broad O-H stretching vibration around 3400 cm^−1^, C-H stretching vibrations around 2900 cm^−1^ and a band around 900–890 cm^−1^ which is associated with the β-glycosidic linkages of the polysaccharide backbone [50].

The incorporation of the active pharmaceutical ingredient in two concentrations does not alter the molecular structure of the polymeric matrix and does not affect the main functional groups of the polymers. This aspect indicates a homogenous distribution of indomethacin into the spongious network, which is essential to obtain a sustained and localized availability of the drug at the lesion area [51].

Overall, FT-IR analysis confirmed that the incorporation of methylcellulose and indomethacin did not disrupt the typical secondary structure of collagen for samples with up to 25% MC gel. The preservation of the characteristic functional groups indicates that the structural integrity of collagen is maintained in samples with 100% collagen gel and in those with the minimum content of MC gel (25%), which is important for preserving the physicochemical and biological properties required for wound dressing applications.

### 2.2. Scanning Electron Microscopy (SEM)

Scanning electron microscopy (SEM) is a commonly used method for evaluating the surface and internal structure of materials, resulting in high resolution images. It provides details about the morphology and porosity of the analyzed samples, which are essential for the characterization of dressings used in the management of skin lesions [52]. To support tissue regeneration, a dressing needs a high porosity that can easily allow the exchange of water vapor and oxygen and that promotes biological fluid absorption and moisture retention. Therefore, its ability to retain exudate at the level of the lesion is closely related to the porosity of the material [53,54]. Moreover, the porous structure promotes nutrient and cell transport while providing a large surface area that favors cell adhesion and migration [55].

**Figure 2:**
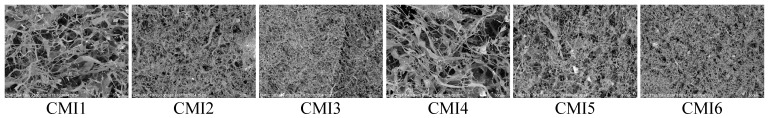
SEM images (×100) of CMI1–CMI6 samples.

**Figure 3:**
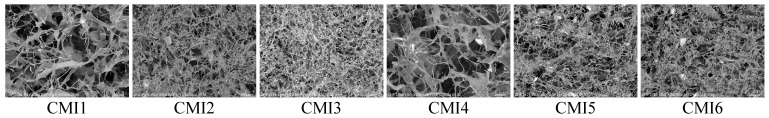
SEM images (×200) of CMI1–CMI6 samples.

Images obtained by SEM analysis, using either a magnification of 100× with a scaling of 500 µm, or a magnification of 200× with a scaling of 200 µm, are shown in Figure 2 and Figure 3.

The images obtained by SEM analysis revealed a series of differences in the internal architecture of the matrices, influenced by the different proportions of polymers used in each formulation.

CMI1 and CMI4 formulations, composed exclusively of collagen and indomethacin, showed a network of collagen fibers, with interconnected pores of variable shapes and sizes, distributed unevenly throughout the matrix, which are typical for native collagen [56]. The high level of porosity gives these matrices a superior capacity to absorb biological fluids, such as exudate from skin lesions, facilitating the tissue regeneration process [57]. This observation is supported by the high values further obtained for CMI1 and CMI4 samples in terms of absorption capacity, results that will be presented in the following subsections. Collagen biomaterials support wound repair by stimulating the activity of cells such as fibroblasts and macrophages. Their porous and interconnected structure also allows collagen sponges to efficiently absorb blood and exudate, helping to control bleeding and promote healing [58].

With the addition of methylcellulose to the samples, significant variations are observed at the morphological level, influenced by the nature of the dominant polymer used. SEM analysis of CMI2 and CMI5 matrices, containing 75% collagen gel and 25% methylcellulose gel, reveals a porous microstructure specific to collagen, but with smaller pores. This feature can be attributed to the presence of parallel-oriented cellulose fibers, which determine a more compact organization of the internal structure [59]. The lower porosity of these matrices is correlated with a reduced absorption capacity compared to that recorded by the formulations based on collagen only (CMI1 and CMI4), the pore size varying inversely proportional to the proportion of MC gel.

Further, increasing the MC gel content to 50% led to the formation of porous matrices with integrated cellulose fibers and a compact structure, with the morphological characteristics of methylcellulose being dominant. Reducing the proportion of collagen led to a decrease in the porosity of the CMI3 and CMI6 matrices, while the parallel, lamellar structure specific to cellulose fibers becomes more prominent due to the higher MC gel content. This internal structure directly influenced the hydration behavior of the formulations, with CMI3 and CMI6 sponges exhibiting the lowest absorption capacity among the samples and rapidly losing structural integrity upon immersion in phosphate buffer.

Regarding the non-steroidal anti-inflammatory drug incorporated into the designed matrices, indomethacin could be observed in all samples, both in free form, on the surface, and embedded in the polymer network, an aspect that has important implications in the mechanism of indomethacin release from the porous matrices.

### 2.3. Compressive Strength Testing

**Figure 4:**
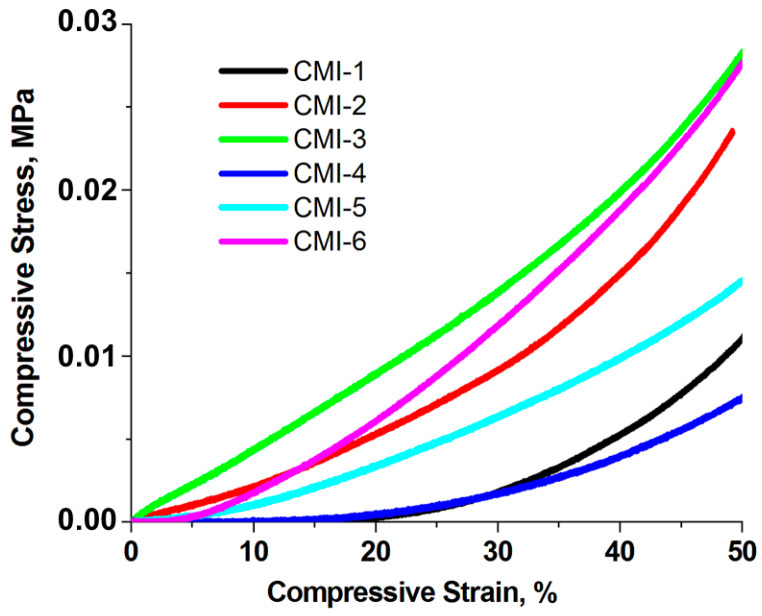
Compression stress/strain curves for CMI1–CMI6 samples.

Mechanical testing is essential for wound dressings to ensure adequate structural stability, flexibility, and resistance during handling and application [60]. Although collagen possesses excellent biocompatibility and bioactivity, its poor mechanical resistance limits its standalone application. Therefore, blending collagen with other polymers represents an effective strategy to improve mechanical performance. In particular, the addition of cellulose derivatives has been reported to enhance its mechanical properties [61,62]. In this regard, compressive strength testing was performed to evaluate the effect of MC incorporation on the mechanical properties of the matrices, and the corresponding stress/strain curves are presented in Figure 4.

The obtained results showed that increasing the methylcellulose content improved the compressive properties of the matrices, likely due to the formation of a denser and mechanically reinforced polymeric network. The highest compressive strength values were observed for CMI3 (0.042 MPa) and CMI6 (0.035 MPa) samples, which also showed higher compressive modulus values (0.14 MPa and 0.12 MPa), indicating that the material becomes stiffer as the methylcellulose content increases. These findings were consistent with the SEM analysis, which revealed a significantly denser and more compact morphology. The increased methylcellulose content likely reinforced the porous network, resulting in superior mechanical resistance. However, excessive structural compactness, although beneficial for mechanical resistance in the dry state, may negatively influence fluid uptake behavior and structural stability under physiological conditions. Moreover, due to the water-soluble nature of methylcellulose, these formulations showed reduced structural stability during absorption and enzymatic degradation studies. In contrast, CMI2 and CMI5 displayed a more balanced architecture, combining adequate mechanical integrity with improved stability under hydrated conditions. This balance may explain their superior in vivo wound healing performance. CMI1 and CMI4 samples exhibited the weakest mechanical performance within their respective series (CMI1–CMI3 and CMI4–CMI6), which is consistent with previous literature reports indicating that the addition of cellulose derivatives enhances collagen’s mechanical properties.

### 2.4. Contact Angle Measurements

Contact angle is a parameter that can be used in the characterization of the wetting behavior of solid surfaces [63]. Thus, the contact angle is an important indicator of the interaction between a liquid droplet and a solid surface, thus providing information about the physicochemical properties of the surface, but also about the chemical composition, hydrophilicity and degree of wetting of the analyzed polymeric support [63,64]. This analysis is often used and is essential for characterizing the wetting behavior of materials with applications in various fields, including the biomedical one [65]. Therefore, hydrophilic polymeric dressings play an important role in the wound healing process by impacting essential properties such as exudate absorption capacity and promoting cell proliferation and adhesion [66].

**Figure 5:**
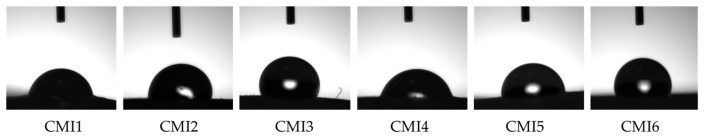
Images of phosphate buffer (pH = 7.4) droplets, in contact with CMI1–CMI6 samples.

**Figure 6:**
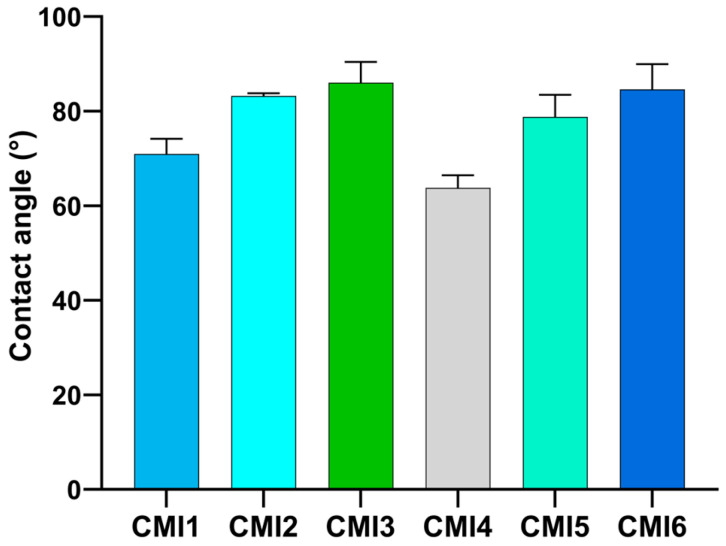
Contact angle values for CMI1–CMI6 samples.

The analysis of the hydrophilicity of designed matrices by determining the contact angle aimed to study the behavior of matrices in contact with the exudate present at the lesion level. Images captured during the contact angle analysis are shown in Figure 5. Mean contact angle values for CMI1–CMI6 samples are presented comparatively in Figure 6.

Analyzing the graph presented above, it can be observed that all samples presented a hydrophilic surface, the contact angle values obtained being less than 90° for all six samples. Within the two series of samples with the same NSAID concentration, CMI1–CMI3 and CMI4–CMI6 respectively, an increase in the contact angle value was observed, correlated with the increase in the percentage of methylcellulose in the sample.

These results can be correlated with the images obtained in the morphological analysis by scanning electron microscopy, where a more compact structure of the polymer network was highlighted, with smaller pores, with the increase in the percentage of methylcellulose in the samples. Also, these results can be correlated with those further obtained in the evaluation of the absorption capacity, which was lower in the case of matrices with a high percentage of methylcellulose.

In addition, doubling the amount of the drug in the matrices from the CMI4–CMI6 (0.2% indomethacin) series led to a slight decrease in the contact angle, compared to the samples with a lower drug content, respectively those from the CMI1–CMI3 series (0.1% indomethacin). Although indomethacin is generally lipophilic, the lower contact angles observed for CMI4–CMI6 compared with CMI1–CMI3 suggest that increasing the drug concentration altered the surface organization of the collagen matrix in a way that favored water spreading. This effect may be related to changes in surface roughness, drug distribution, or exposure of polar groups within the collagen network. Therefore, the wettability of these formulations is not governed only by the intrinsic hydrophobicity of indomethacin, but also by its influence on matrix architecture and surface chemistry [67].

The hydrophilic character and wettability of the developed collagen–methylcellulose matrices are consistent with previously reported collagen-based wound dressings, where low contact angle values were associated with improved exudate absorption, cell adhesion, and maintenance of a moist environment favorable for tissue regeneration. Fabric-like bacterial cellulose/collagen membranes developed for wound dressing applications exhibited highly hydrophilic surfaces, with water contact angles below 15° within the first second, together with high absorbent capacity for wound exudates. These properties were considered favorable for maintaining a moist wound environment and promoting tissue repair [68]. A 3D polycaprolactone/collagen nanofibrous dressing designed for diabetic wound healing demonstrated high swelling and water absorption capacities, characteristics associated with improved exudate management and enhanced cellular infiltration within the scaffold structure [69]. Aloe vera-loaded zein/polycaprolactone/collagen nanofibrous scaffolds showed improved hydrophilicity, as reflected by reduced water contact angle values with a decreasing zein/polycaprolactone ratio. The enhanced wettability was correlated with improved fibroblast adhesion and favorable properties for wound healing applications [70].

The contact angle measurements indicate that all developed matrices remain overall hydrophilic, with values ranging from 63.77° to 86.03°, while also suggesting a gradual increase in hydrophobicity with higher methylcellulose content. This confirms that MC incorporation enables controlled modulation of surface wettability while preserving a hydrophilic character. Such modulation is relevant in the context of wound healing, where maintaining an optimal moisture balance is essential. Wound exudate plays an important role in tissue repair: insufficient moisture can cause scarring and delay healing, whereas excessive exudate may lead to maceration and increased infection risk [71]. In this regard, a slight reduction in surface hydrophilicity may be beneficial, as it can help prevent excessive fluid uptake, thereby contributing to more controlled exudate management, while still preserving sufficient wettability to support cell adhesion and proliferation. Therefore, the observed range of contact angle values suggests a balanced wetting behavior that may be favorable for wound dressing applications, where both moisture regulation and biocompatibility are required. Among the tested formulations, the more hydrophilic collagen-based samples (CMI1 and CMI4) may favor rapid protein adsorption and cell attachment [72], whereas MC-containing matrices may provide a more balanced interface that better regulates fluid uptake and supports sustained tissue regeneration.

### 2.5. Evaluation of the Absorption Capacity

Absorption capacity is an important quality attribute in the development of effective dressings for the treatment of skin lesions [73]. Optimal values obtained in this analysis can be correlated with adequate absorption of exudate from the lesion, without causing wound dehydration [73]. In addition, absorption capacity has implications in the release process of the drug incorporated into the polymer network, controlling its diffusion [74]. Collagen-based sponges possess a high capacity to absorb wound exudate, contributing to moisture balance and improved healing conditions [75].

**Figure 7:**
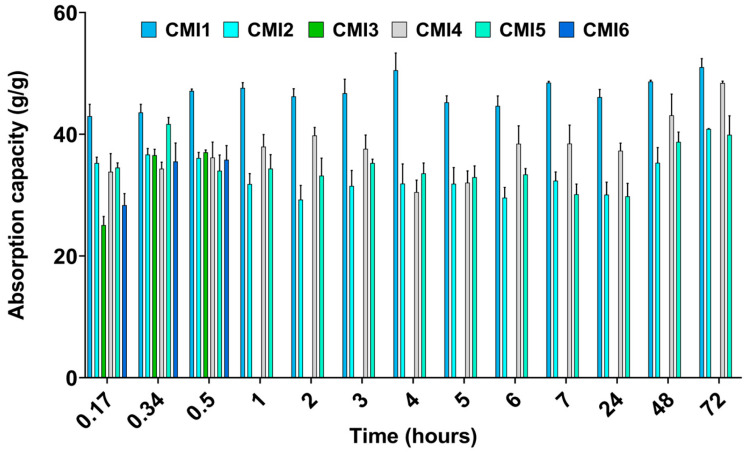
Absorption capacity of CMI1–CMI6 samples at different time intervals.

The absorption capacity of CMI1–CMI6 sponge-like matrices was investigated using a gravimetric method, and the results obtained at different time points are presented in Figure 7.

The highest absorption capacity was recorded in the case of CMI1 (51.009 g/g) and CMI4 (48.418 g/g) samples, respectively, both having 100% collagen gel in their composition, followed by matrices CMI2 (40.873 g/g) and CMI5 (39.906 g/g), with a collagen gel content of 75% and 25% methylcellulose gel. These results can be correlated with the porous structure highlighted in the morphological analysis by scanning electron microscopy.

The addition of methylcellulose to the composition of the matrices led to a gradual decrease in absorption capacity, with the matrices with the highest methylcellulose content (CMI3 and CMI6) rehydrating and transforming into a gel after 60 min of contact with phosphate buffer. Although MC is a polymer with suitable properties for use in the development of modern dressings, its high solubility in water [18] can lead to rapid destruction and, therefore, to a faster release of the drug substance. Thus, matrices containing a large amount of MC quickly lost their structural integrity in contact with phosphate buffer (pH 7.4) solution. Doubling the amount of anti-inflammatory drug led to a decrease in the absorption capacity, a phenomenon observed in the case of CMI1 and CMI4 samples. Indomethacin is a chemical compound with a hydrophobic character [76]. Thus, it is possible that it interacts with the hydrophilic structure of collagen and influences the absorption capacity of collagenic matrices. Regarding matrices with a collagen: MC ratio of 3:1 (CMI2 and CMI5), the effect of the drug on the absorption capacity of the samples was not as obvious.

Comparing the two series of sponge-like matrices, a decrease in absorption capacity is observed that varies inversely proportionally with the increase in the amount of methylcellulose in the sample. For the CMI1–CMI3 series, the decrease in absorption capacity occurred in the order CMI1 > CMI2 > CM3, while for the CMI4–CMI6 series, the results varied as follows: CMI4 > CMI5 > CMI6. The CMI1, CMI2, CMI4 and CMI5 matrices with a collagen percentage of at least 75% presented the characteristic absorption behavior of collagen, starting from an initial phase of rapid liquid absorption, followed by reaching a relatively stationary, equilibrium level, in which the absorption capacity of the matrices did not change significantly [77]. The swelling behavior of the developed collagen–methylcellulose matrices is comparable to previously reported collagen-based wound dressings, where moderate-to-high swelling capacity was associated with efficient exudate absorption, moisture retention, and maintenance of a favorable environment for wound healing. Thus, collagen/microfibrillated carboxymethylcellulose blend films designed as wound dressings exhibited a mild swelling behavior, with swelling values reaching approximately six-fold after 24 h. The controlled swelling profile was considered advantageous for maintaining structural integrity while absorbing wound exudates [62]. Collagen/hyaluronic acid biodegradable wound dressings loaded with antibacterial agents demonstrated superior swelling properties, which were attributed to the hydrophilic nature of collagen and hyaluronic acid networks. Enhanced swelling was associated with improved fluid uptake and maintenance of a moist wound environment favorable for healing [78]. Fish collagen–hyaluronate composite lyophilized scaffolds modified with sodium alginate showed high water absorption capacities ranging from 380% to 1382% and equilibrium water contents between 79% and 94% after 24 h incubation. The elevated swelling and water retention abilities were considered beneficial for managing highly exuding chronic wounds [79].

**Figure 8:**
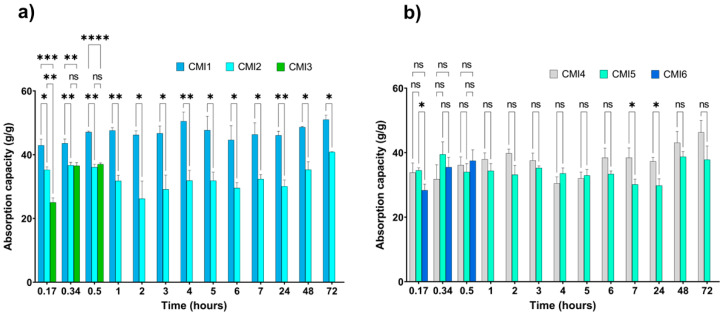
Absorption capacity (g/g) variation for: (**a**) CMI1, CMI2, CMI3 and (**b**) CMI4, CMI5, CMI6. Statistical significance was assessed by two-way ANOVA with Tukey’s multiple comparisons test: * *p*  <  0.05; ** *p*  <  0.01; *** *p*  <  0.001; **** *p*  <  0.0001; ns: not significant.

Statistical analysis of the absorption behavior was further performed to evaluate differences in equilibrium swelling among formulations. As shown in Figure 8a, a statistically significant difference was observed at 72 h between CMI1 and CMI2 matrices (*p* < 0.05), indicating that the collagen/methylcellulose ratio significantly influenced equilibrium swelling in the formulations containing 0.1% indomethacin.

In contrast, no statistically significant difference was identified at 72 h between CMI4 and CMI5 (Figure 8b, *p* > 0.05). For this group, significant differences were detected only at the earlier time points of 7 h and 24 h (*p* < 0.05), whereas the remaining comparisons were not statistically significant. As mentioned above, due to hydrogel formation and loss of structural integrity, CMI3 and CMI6 could not be reliably evaluated under equilibrium swelling conditions at 72 h and were therefore excluded from the final statistical comparison. In addition, formulations with identical polymeric composition but different indomethacin loading (CMI1 vs. CMI4, CMI2 vs. CMI5, and CMI3 vs. CMI6) were also statistically compared. However, these comparisons did not show statistically significant differences (*p* > 0.05).

### 2.6. In Vitro Enzymatic Degradation

In vitro enzymatic degradation is a parameter that characterizes the stability of dressings when they come into contact with the wound site [80]. The collagenase test is part of the enzymatic degradation assay, being used in vitro to simulate and characterize the enzymatic decomposition of the material. Collagenase, which is part of the matrix metalloproteinase (MMP) group, is an enzyme commonly found at wound level, and the degradation induced by it is associated with a loss of mechanical strength. Therefore, a material that shows a lower degradation rate is associated with more mechanical stability, an important factor for the characterization of modern dressings [80,81]. In addition, collagen-based dressings have become frequently researched and used, as they have been demonstrated to lower elevated MMP levels, which can be correlated with a low healing rate, and act as a perfect substrate for proteases, preventing the transformation of the wound into a chronic wound. Thus, collagen-based dressings, applied early in the injury process, have a significant impact on healing rates and provide a temporary skin matrix [82].

**Figure 9:**
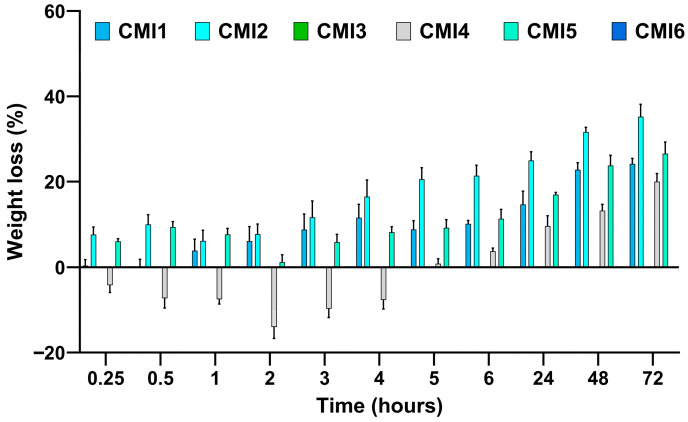
Weight loss recorded by CMI1–CMI6 samples.

The degree of enzymatic degradation of the sponge-like matrices was assessed by monitoring their weight loss over a given time interval. The weight loss of CMI1–CMI6 samples over a 72 h period is shown in Figure 9.

Following the analysis of the data presented in the graphic above, it can be concluded that collagen matrices with 100% collagen gel (CMI1 and CMI4) and the samples with a content of 25% methylcellulose gel (CMI2 and CMI5) were stable in contact with the collagenase solution (37 °C) throughout the experiment. Regarding samples CMI3 and CMI6, with a methylcellulose gel content of 50%, they were transformed into hydrogel in the soaking stage, preceding the analysis, the results correlating with those obtained in the absorption capacity analysis, where the matrices CMI3 and CMI6 with 50% methylcellulose gel quickly lost their integrity in contact with the phosphate buffer solution.

CMI4 and CMI5 matrices, with double drug concentration, degraded more slowly compared to CMI1 and CMI2 matrices. The maximum weight loss recorded 72 h after the start of the experiment was 20% and 26.55% for CMI4 and CMI5 samples, while CMI1 and CMI2 matrices recorded a 24.17% and 35.24% weight loss, respectively. Matrices with a high collagen content (CMI1 and CMI4) underwent slower degradation in the presence of the collagenase solution, of 24.17% and 20%, respectively, compared to matrices with a 25% methylcellulose gel content (CMI2 and CMI5), which had a maximum weight loss of 35.24% and 26.55%, respectively. This phenomenon can be explained by the solubility of the cellulose derivative in aqueous solutions, matrices with 25% methylcellulose gradually transforming into the corresponding hydrogels and thus registering a weight loss, independent of that due to collagenase activity.

For CMI1–CMI3 matrices, containing the same amount of the drug but different proportions of polymers, the maximum weight loss recorded occurred as follows: CMI2 > CMI1. Since the CMI3 matrix transformed into a hydrogel in the pre-analysis stage (24 h in phosphate buffer to reach the equilibrium stage), it could not be directly compared with the other two matrices. In contrast, in terms of the stability of the matrices CMI1 and CMI2, sample CMI1 presented the lowest degradation rate, indicating a higher stability compared to CMI2, with 25% methylcellulose gel, a phenomenon possibly attributed to the water solubility of the biopolymer.

CMI4–CMI6 samples presented similar results compared to CMI1–CMI3 matrices. At the composition level, CMI4–CMI6 samples presented double the amount of the drug compared to CMI1–CMI3. The maximum weight loss recorded occurred as follows: CMI5 > CMI4. Like the CMI3 matrix, the CMI6 matrix transformed into a hydrogel in the soaking stage, both having a methylcellulose gel content of 50%. Thus, the CMI6 matrix could not be properly included in the comparative analysis of stability in the presence of collagenase. Therefore, the resistance to enzymatic degradation of the CMI4 and CMI5 matrices was compared, with the CMI4 sample (composed entirely of collagen gel) showing the lowest degradation rate.

### 2.7. In Vitro Release Kinetics of Indomethacin from the Sponge-like Matrices

The ability to release the active substance in a controlled manner from porous matrices is relevant for obtaining an effective therapeutic response that contributes to wound healing [51]. Several factors are involved in the controlled release process of the drug from different pharmaceutical forms, such as: the physicochemical properties of the active substance and polymers, the design of the pharmaceutical formulation and degradation behavior of the polymeric support [83,84,85]. The importance of studying the drug release pattern from pharmaceutical formulations is relevant to demonstrate the efficacy and safety of the formulation [86]. Sponge-like matrices composed of collagen, methylcellulose and indomethacin, in different proportions, which were subsequently cross-linked with glutaraldehyde, were investigated in terms of in vitro release kinetics.

**Figure 10:**
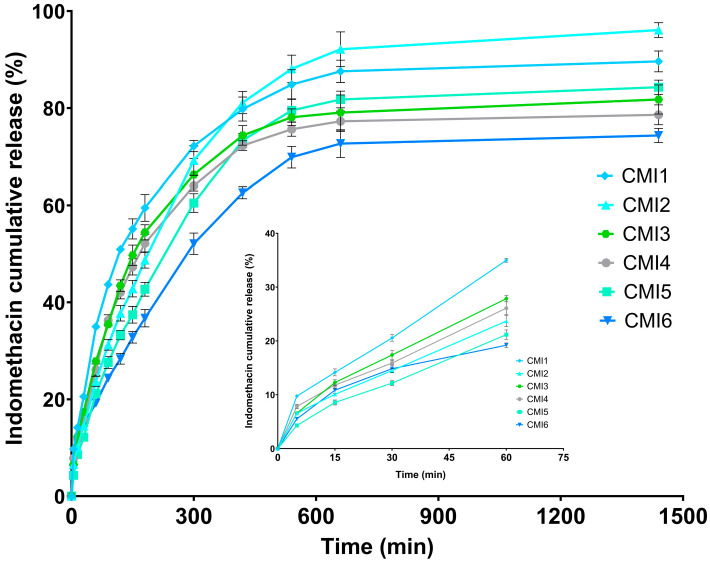
Evolution of in vitro release of indomethacin from CMI1–CMI6 samples over a 24 h period.

To evaluate and compare the mechanism of indomethacin release from the designed polymeric systems, the corresponding kinetic profiles were plotted, graphically representing the cumulative percentage amount of indomethacin released from the polymeric matrices, as a function of time. The kinetic profiles obtained for CMI1–CMI6 matrices are presented in Figure 10, highlighting the initial 60 min interval.

The analysis of the kinetic release profiles of indomethacin from CMI1–CMI3 sponge-like matrices, which contain the same drug concentration (0.1%), but different proportions of the two biopolymers, highlights significant variations in the cumulative percentage amount released within 24 h. Thus, the values range between 81.78% for CMI3 and 96.09% for CMI2, which reflects the influence of the composition of the polymeric supports on the release mechanism. In this regard, an increase in the proportion of methylcellulose gel led to a decrease in the cumulative amount (%) released from the obtained matrices. This decrease—by 7.85% for CMI3 compared to CMI1, and by 14.31% compared to CMI2—can be attributed to the more compact microstructure of the CMI3 matrix, highlighted in the morphological analysis by SEM. Within this series of samples, the highest cumulative released amount (%) was recorded for the CMI2 matrix, with a methylcellulose gel content of 25%. Thus, it is possible that a 3:1 collagen–MC ratio leads to the obtaining of an intermediate microstructure, with a porosity and a fiber network that favors the diffusion of the active substance.

Analyzing the initial period of the experiment (first 60 min), according to Figure 10, all matrices recorded a rapid release of indomethacin, a phenomenon known as burst release. The percentage values varied between 23.69% for CMI2 and 34.99% for CMI1. After this initial phase, the process continues at a moderate rate, with a gradual increase in the cumulative amount released, followed by a plateau phase in which the release rate stabilizes. The burst release phenomenon highlighted in the first part of the analysis can be attributed to the presence of indomethacin crystals on the surface of the matrices, confirmed by SEM imaging (Figure 2 and Figure 3) [87]. At the same time, the slower release of the drug from CMI2 and CMI3 matrices in the first 60 min could be correlated both with the more compact structure of the matrix and with the possible increase in the viscosity of the hydrated system.

Regarding the CMI4–CMI6 matrices, which presented the same polymer composition as CMI1–CMI3 samples, but a double drug concentration (0.2%), the kinetic profiles indicate that these samples presented a lower cumulative percentage released at the end of the experiment (24 h), between 84.29% for the CMI5 matrix and 74.34% for the CMI6 matrix. Thus, the same trend was observed as in the case of the CMI1–CMI3 samples, with a difference of 4.27% between CMI4 and CMI6 and of 9.95% between CMI5 and CMI6. At the same time, the matrix that recorded the highest released amount (%) of indomethacin was the CMI5 matrix, with a 3:1 collagen–MC ratio. In the first 60 min, the percentage release amounts were between 19.21% for the CMI6 matrix and 26.10% for the CMI4 matrix, confirming the presence of a less pronounced burst release effect in the case of increased indomethacin concentration. In addition, the CMI5 matrix recorded an intermediate value, while the CMI6 matrix with 50% MC gel showed the least pronounced burst release effect. Comparing CMI2 and CMI5 matrices, which contain the same collagen:MC gels ratio (75:25), but different amounts of the drug (0.1% vs. 0.2%), the release profile indicates a significantly higher cumulative release for CMI2 (96.09%), compared to CMI5 (84.29%) at 24 h, with a difference of 11.80%. This behavior is also reflected in the initial stage: 23.69% for CMI2 and 21.17% for CMI5, respectively, in the first 60 min. According to Figure 10, the descending order of cumulative indomethacin released after 24 h was as follows: CMI2 > CMI1 > CMI5 > CMI3 > CMI4 > CMI6.

Regarding the data available in the scientific literature, several porous wound dressing systems based on collagen and/or polysaccharides have demonstrated biphasic drug release profiles characterized by an initial burst release followed by a sustained release phase. Afzali et al. [88] developed alginate–collagen–hyaluronate composite systems that exhibited an initial rapid drug release during the first hour, followed by controlled release over 72 h. Similarly, collagen–dextran spongious wound dressings loaded with flufenamic acid showed burst release values between 29.97% and 46.37% within the first hour and cumulative drug release reaching approximately 95% after 10 h [89]. Comparable biphasic behavior was reported for collagen–hydroxyethylcellulose sponges containing naproxen, where the initial burst release ranged from 14.93% to 30.89% after 60 min and cumulative release values varied between 62.24% and 96.12% after 24 h [51]. Similar release mechanisms were also observed for sodium alginate sponges loaded with ibuprofen [90] and freeze-dried carboxymethylcellulose/chitosan composite sponges containing gentamicin, where release kinetics were strongly influenced by polymer composition, drug hydrophilicity, and pH [91].

The collagen–MC spongious matrices developed in the present study exhibit release characteristics that are consistent with this class of conventional sponge-like wound dressing systems. The initial burst release observed during the first 60 min (19.21–34.99%) and the cumulative indomethacin release after 24 h (74.34–96.09%) fall within the ranges reported for similar porous systems, supporting the applicability of these matrices for controlled topical drug delivery. Like previously reported collagen–polysaccharide sponges, the biphasic release profile identified in this study is mainly governed by rapid dissolution of the surface-retained drug followed by diffusion through the hydrated porous network. Moreover, the modulation of the release rate with increasing methylcellulose content is consistent with literature findings describing the influence of polymer composition and matrix compactness on diffusion-controlled transport.

Recent advances in wound dressing design have focused on more structurally complex collagen–cellulose composite systems aimed at further improving release control. Collagen–cellulose nanofiber composite aerogels reinforced with tannic acid [92] demonstrated that increasing network organization modulates drug release kinetics within a similar overall timeframe (~10 h). Collagen-only aerogels exhibited rapid release, reaching approximately 95% within 2 h, while the incorporation of cellulose nanofibers reduced the initial burst, achieving ~80% release at 4 h. In contrast, tannic acid reinforcement resulted in a more sustained and gradual release profile, reaching approximately 80% at 10 h. This behavior highlights the influence of aerogel-type highly porous architectures on diffusion kinetics, where network reinforcement mainly affects early-stage release. In comparison, the collagen–MC matrices developed in this study, based on a conventional sponge-like structure, provide a more extended biphasic release over 24 h. This difference may be related to distinct mass transfer characteristics, with hydrated sponge systems supporting more sustained diffusion through fluid uptake and swelling. Similarly, biohybrid cellulose acetate–collagen bilayer wound dressings incorporating either bioactive latex or ciprofloxacin [93] exhibited an initial burst release of approximately 18–19%, followed by sustained release up to 72 h due to the combined effect of nanofibrous and porous layers regulating swelling and diffusion. A multifunctional bilayer scaffold based on bacterial cellulose/polyvinyl alcohol fibers and collagen/polyvinyl alcohol hydrogels [94] also showed composition-dependent sustained curcumin release over 32 h, strongly influenced by crosslinking density, swelling, and intermolecular interactions. In comparison, while these advanced systems provide extended-release profiles through engineered multilayer architectures or nanofiber reinforcement, the collagen–methylcellulose matrices developed in this study achieve a balanced biphasic release over 24 h using a simpler porous sponge-like structure.

Regarding indomethacin delivery systems, this drug has been incorporated into various topical formulations, including porous collagen/polyvinyl alcohol matrices, in situ gels, and polysaccharide-based films. Collagen/PVA matrices showed a biphasic mechanism of indomethacin release with cumulative drug release ranging from 82.28% to 97.34% over 24 h [31], whereas poloxamer–hyaluronic acid in situ gels exhibited slower, sustained release profiles with approximately 59.75% of the drug released over 24 h [27]. Pectin-based films loaded with indomethacin showed rapid release, with more than 50% of the drug released within 120 min following diffusion and erosion-controlled kinetics [29]. These findings further confirm that indomethacin release is highly dependent on matrix composition and structural organization.

To investigate the mechanism of indomethacin release from collagen–MC sponge-like matrices, several kinetic models were used, including the Power law, the Higuchi, and the zero-order models (Equation (3)). The quality of the fit was assessed by both the correlation coefficient (R) and the adjusted coefficient of determination (adjusted R^2^), an essential parameter in comparative analysis, as it integrates the influence of the number of parameters and penalizes excessively complex models. In this context, the use of adjusted R^2^ allows a more objective assessment of the performance of the models, being particularly useful in the comparison between the Power law model, which includes the release exponent *n*, and the Higuchi model, which is characterized by a more simplified formulation [95]. The values of the statistical parameters obtained for the three kinetic models analyzed are given in Table 1, along with the characteristic parameters of the Power law model.

As indicated in Table 1, the Power law model yielded the highest values for both the correlation coefficient and the adjusted coefficient of determination, demonstrating its superior ability to describe the experimental release profiles. Specifically, R values ranged between 0.9601 and 0.9663. Compared to the Higuchi model, the Power law model consistently showed improved statistical performance, supporting its suitability for characterizing the release mechanism of indomethacin from collagen–MC sponge-like matrices, despite its increased mathematical complexity.

To further refine model selection, the Akaike Information Criterion (AIC) was employed, as it enables a more comprehensive evaluation by accounting for both the goodness-of-fit and the number of model parameters. Unlike R and adjusted R^2^, which primarily assess fitting accuracy, AIC provides insight into the trade-off between model precision and complexity. Considering the limited number of experimental data points, the corrected form of the criterion (AIC_c_) was applied to ensure a more reliable comparison [96].

The results presented in Table 1 show that the lowest AIC_c_ values correspond to the Power law model, indicating that it achieves the most favorable balance between accuracy and precision [97]. Moreover, the difference in AIC_c_ values between the Power law and the Higuchi model exceeded the threshold of 2, further supporting the superior descriptive capability of the Power law approach [98].

Overall, the combined evaluation of all statistical parameters confirms that the Power law model provides the most appropriate framework for describing the release kinetics of indomethacin from collagen–MC sponge-like matrices. Furthermore, release exponent (n) values below 0.5 indicate a deviation from classical Fickian diffusion, suggesting a complex drug release mechanism which is characteristic of porous matrices, as we have shown in our previous studies [31,51,89]. Initially, rapid wetting of the matrices’ surface occurs upon contact with the wound exudate, leading to the release of the weakly bound drug from superficial regions. This stage can be associated with the burst release effect. Subsequent fluid absorption and penetration into the porous network led to hydration and swelling of the polymer structure. As the matrices become increasingly hydrated, the drug diffuses progressively through the swollen network, while a slower release phase is controlled by the drug diffusion entrapped into the spongious structure together with gradual polymer erosion.

In conclusion, the collagen–methylcellulose sponge-like matrices exhibit a biphasic release behavior characterized by an initial burst release followed by sustained indomethacin delivery over 24 h. The release performance is governed by polymer composition and drug loading, which modulate both early and prolonged release phases. While more complex systems may achieve extended release profiles, the present formulation provides a clinically relevant therapeutic window using a simple and easily adjustable polymeric platform, making it suitable for early-stage wound healing applications requiring rapid onset and sustained anti-inflammatory action [88].

### 2.8. Evaluation of Wound Healing in Experimental Animal Models

Burn wound healing is a highly dynamic process involving four overlapping phases—hemostasis, inflammation, proliferation, and tissue remodeling—each activated at specific times and with varying intensity depending on local and systemic factors. Early events, particularly tissue homeostasis and post-traumatic inflammation, play a crucial role in guiding subsequent tissue repair and remodeling, making them primary targets for effective burn treatment. During this stage, activation of inflammatory signaling pathways leads to elevated levels of pro-inflammatory cytokines, which promote neutrophil migration and macrophage activation [31].

**Figure 11:**
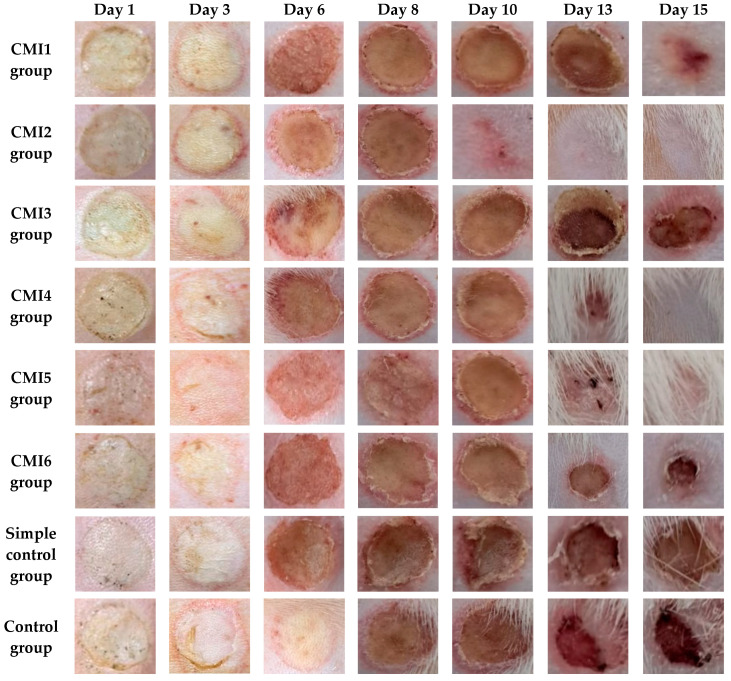
Representative images of burn wound evolution in Wistar rats treated with CMI1–CMI6 porous matrices, simple collagen matrix (drug-free control group) and gauze (control group), captured at different time intervals (day 1 defined as t = 0).

The designed porous matrices were evaluated in vivo on Wistar rats with experimentally induced burns to evaluate their wound-healing performance and potential regenerative effects. As described in the Materials and Methods section, the animals were divided into eight groups as follows: the control group was treated with sterile gauze, the simple control group with collagen sponge, while the other groups received the six different sponge-like matrices based on collagen, MC, and indomethacin, named CMI1–CMI6. Figure 11 presents the progression of the induced burns on animal models. Immediately after the burn induction, lesions were characterized by the presence of a white eschar, indicating an impairment of both epidermal and dermal layers, surrounded by a hyperemic zone. This hyperemia reflects increased blood flow and vasodilation triggered by cellular disruption, marking the onset of the inflammatory response, which is evident from the first day [99].

The inflammatory phase begins within the first hours following skin injury and serves as an essential defense mechanism against microbial invasion at the lesion site. However, this healing stage is also associated with elevated levels of pro-inflammatory cytokines, which can delay the healing process [100].

Despite this response, inflammation represents a critical stage in tissue repair and constitutes a primary therapeutic target in wound management. In this context, the short-term topical application of non-steroidal anti-inflammatory drugs has been shown to effectively reduce inflammation and pain, thereby promoting and accelerating tissue re-epithelialization [20].

Although indomethacin is known to modulate key inflammatory mediators such as TNF-α and IL-6, this study was primarily designed to investigate wound healing at the macroscopic level, using wound size reduction as an indicator of tissue regeneration. The localized release of the anti-inflammatory agent from the collagen–methylcellulose-based matrices can contribute to the modulation of the early inflammatory phase, thereby facilitating progression toward the proliferative stage of tissue repair.

The wound healing process was significantly accelerated in the groups treated with CMI1–CMI6 matrices compared to the control and simple control groups. Early effects were observed by the third day, when the matrices effectively absorbed the exudate from the burn injuries and initiated re-epithelialization. As healing progressed, the formation of a crust became evident by day 6 and was fully established by day 8 in all groups, although the process was more advanced in the collagen–MC sponge-like matrices-treated groups. On day 13 of observation, the animals treated with the CMI2 matrix were completely healed, while the animals which received the other five matrices had a healing process over 80% and over 85% by the end of the observation period (day 15).

**Figure 12:**
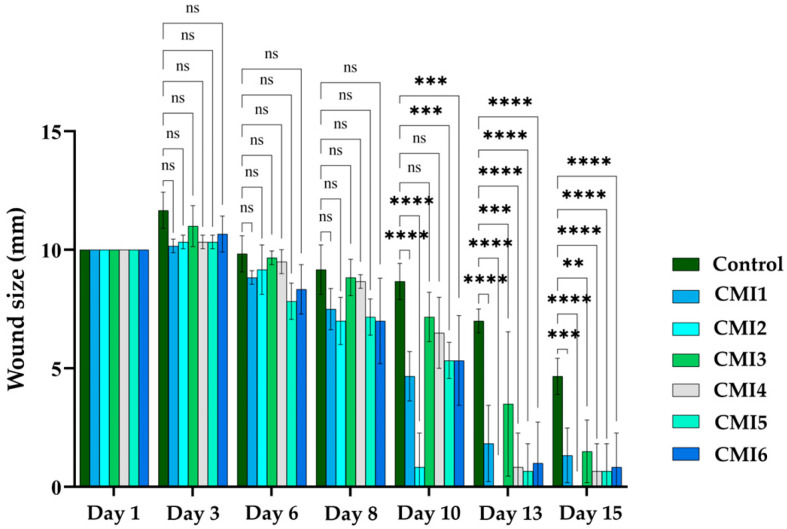
Changes in wound size (mm) over a 15-day period following treatment with CMI1–CMI6 porous matrices compared to gauze (control). Statistical significance was determined using ANOVA with Dunnett’s multiple comparison test (vs. control): ** *p*  <  0.01, *** *p*  <  0.001, **** *p*  <  0.0001; ns: not significant.

**Figure 13:**
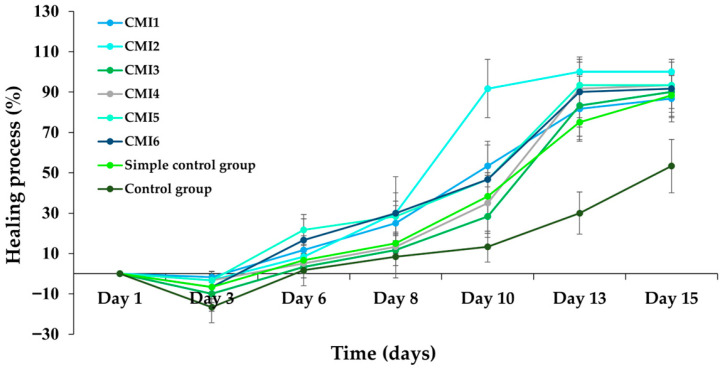
Progression of the burn healing process (%) over 15 days in Wistar rats treated with CMI1–CMI6 porous matrices, collagen matrix (simple control), and gauze (control).

Figure 12 shows the progression of wound size (mm), while Figure 13 illustrates the corresponding healing evolution, calculated through Equation (4).

At 3 days post-burn, a mild post-traumatic inflammatory response was observed for all groups, which was associated with a slower healing process (Figure 12 and Figure 13).

After 6 days, a reduction in wound size was observed in animals treated with all sponge-like matrices, with values higher than 10% registered for the groups to which CMI1 (100% collagen gel and 0.1% indomethacin), CMI5 (75% collagen gel, 25% MC gel, and 0.2% indomethacin), and CMI6 (50% collagen gel, 50% MC gel, and 0.2% indomethacin) were applied, with a decrease in the lesion diameter of 10.17% for CMI1, 20.34% for CMI5, and 15.25% for CMI6, compared to the control group. For other animals treated with CMI2 (75% collagen gel, 25% MC gel, and 0.1% indomethacin), CMI3 (50% collagen gel, 50% MC gel, and 0.1% indomethacin), and CMI4 (100% collagen gel and 0.2% indomethacin), the decrease in the wound size was slower than 10%, as follows: 6.71% for CMI2, 1.63% for CMI3, and 3.36% for CMI4 in comparison with the control group. At this stage, wounds in the groups treated with all six matrices formed a scab at the injury site, accompanied by a color change to brown, whereas lesions in the control group exhibited a white appearance. By day 8, wound size in the groups of Wistar rats treated with CMI1, CMI2, CMI5, and CMI6, where the reduction was higher than 18% compared to the control group, indicated an accelerated healing process. For CMI3 and CMI4 groups, the decrease was slower than 6% compared to the group treated with sterile gauze.

As can be observed in Figure 11, Figure 12 and Figure 13, on the 10th day of observation, the burn injury size reduced significantly (*p* < 0.0001), with a percentage higher than 90% for CMI2 (90.43%), followed by CMI1 (46.14%, *p* < 0.0001), CMI5 and CMI6 respectively, with a decrease in the wound size of 38.52% in comparison with the control one (*p* < 0.001). For the animals treated with CMI3 and CMI4, the decrease in wound size was lower than the results registered for other matrices: 17.30% for CMI3 and 25.03% for CMI4.

On the 13th day, the scab of wounds for the animals treated with the CMI2 sample fell off, suggesting that the Wistar rats had completely healed (*p* < 0.0001). For these animals, the healing process was considered finished after 13 days of observation (*p* < 0.0001). The healing evolution was also significantly higher for CMI4, CMI5, and CMI6, with percentages greater than 85%: 88.14% (CMI4), 90.43% (CMI5), and 85.71% (CMI6), compared to the control group (*p* < 0.0001), indicating a near-complete stage of tissue repair. For CMI1 and CMI3, the decrease in wound diameter was a little bit lower in comparison with other matrices: 73.86% for CMI1 and 76.14% for CMI3.

On the last day of observation (day 15), it can be observed from Figure 13 that the healing process registered for CMI3–CMI6 groups was higher than 90%, while for the Wistar rats from the CMI1 group, the healing process was 86.67%. The healing was significantly higher than the one recorded for the control group (*p* < 0.01–0.0001).

The wound healing performance of the developed collagen–methylcellulose sponge-like matrices is consistent with recent studies on advanced biopolymeric wound dressings combining collagen with cellulose-based components or similar polymers. Composite systems incorporating collagen and nanocellulose have demonstrated accelerated healing in animal models, achieving up to ~95–99% wound closure within 14–18 days, along with enhanced re-epithelialization, collagen deposition, and neovascularization [101]. Similarly, cellulose derivative-based systems, such as methylcellulose-containing composites, have shown high wound contraction rates (up to ~96–98%) and improved tissue regeneration, supported by reduced inflammation and increased angiogenesis [34,35]. Other hybrid scaffolds combining collagen with polysaccharides, including bacterial cellulose or chitosan, have also exhibited improved healing kinetics, biocompatibility, and structural support for tissue repair in vivo [102,103]. Naproxen-loaded collagen–hydroxyethylcellulose sponge-like matrices significantly improved burn wound healing in Wistar rats, achieving accelerated wound closure, reduced inflammation, and enhanced tissue regeneration compared to control and commercial dressings. The optimized formulations demonstrated superior healing performance over 17 days, highlighting the synergistic effect of the polymeric scaffold and localized anti-inflammatory drug delivery [51].

All animals included in the groups treated with CMI1–CMI6 sponge-like matrices showed significantly accelerated re-epithelialization compared to the control group. The superior therapeutic efficacy of these porous scaffolds can be attributed to the synergy between the biopolymer matrix, collagen and MC, and the incorporated anti-inflammatory agent, indomethacin, which simultaneously acts on the lesional microenvironment, and the inflammatory processes involved in healing.

Glutaraldehyde was employed at a low concentration (0.05%) as a crosslinking agent to stabilize the collagen-based network through reactions with available amine groups, resulting in the formation of covalent linkages. Under these conditions, the majority of the crosslinker is expected to participate in the crosslinking process, thereby limiting the presence of unreacted species in the final sponges.

The crosslinking protocol was designed to favor its incorporation into the polymeric matrix, contributing to the structural stability of the system upon contact with wound exudate. Importantly, the in vivo evaluation did not reveal any signs of local adverse reactions, such as inflammation, irritation, or delayed healing. On the contrary, all treated groups exhibited progressive wound closure, and complete healing was observed within the experimental period, accompanied by normal tissue re-epithelialization, supporting the potential suitability of the developed sponge-like matrices for wound healing applications. Although the in vivo findings are promising, several limitations of the present study should be considered. The evaluation of wound healing was mainly based on macroscopic inspection and measurement of wound area. While this non-invasive methodology enables longitudinal monitoring of the same animals over time, it does not provide detailed insight into the underlying biological and structural processes involved in tissue repair. Therefore, future studies should include comprehensive histological and histochemical assessments—such as hematoxylin and eosin (H&E) and Masson’s trichrome staining—to better characterize inflammatory responses, collagen deposition and organization, re-epithelialization, and tissue remodeling. Another limitation of the present study concerns the lack of in vitro cytotoxicity evaluation for the final formulations. Although collagen–MC matrices with similar compositions were previously shown to be cytocompatible with human fibroblasts [17], the incorporation of a crosslinking agent and a bioactive drug may influence the biological response. Nevertheless, the favorable in vivo wound healing outcomes observed in the present study, without signs of adverse local tissue reaction, support the overall biocompatibility of the developed systems within the scope of this work. However, additional in vitro studies are required to fully characterize their cytotoxic profile.

## 3. Materials and Methods

### 3.1. Materials

The collagen hydrogel used in this study was obtained according to the collagen gel preparation method developed by the Collagen Department, Division of Leather and Footwear Research Institute (ICPI), National Research and Development Institute for Textiles and Leather, Bucharest (INCDTP) [18]. The collagen used was extracted from calfskin, following various acid-base treatments. Methylcellulose was purchased from Janssen Chimica, Beerse, Belgium, indomethacin was from Fagron, Bucharest, Romania, and collagenase was obtained from Merck, Darmstadt, Germany. Glutaraldehyde was employed as a crosslinking agent due to its high efficiency in stabilizing collagen-based matrices through covalent interactions with free amine groups. This process enhances the mechanical strength, structural stability, and resistance to enzymatic degradation of the resulting scaffolds, which are critical properties for wound dressing applications [47,104]. Glutaraldehyde is a well-established crosslinking agent that has been incorporated into commercially available biomedical products, such as Floseal Hemostatic Matrix. Furthermore, the existing literature indicates that, at appropriately controlled concentrations, glutaraldehyde can be employed without inducing significant cytotoxicity, highlighting its potential for safe use in biomaterial design [105,106,107,108,109]. All other reagents were of analytical grade, and the water used was ultrapure.

### 3.2. Preparation of Plain Hydrogels

The fibrillar type I collagen gel used for matrix preparation, with an initial acidic pH of approximately 2 and a concentration of 2.4%, was adjusted to pH values between 7.2 and 7.4 and a concentration of 1% by the controlled addition of 1 M sodium hydroxide solution under mechanical stirring. To obtain the 3% MC hydrogel, the water required for preparation was divided into two equal parts: half was cooled in the refrigerator, and the other half was heated to 80 °C in a water bath. After reaching the desired temperature, the container with the hot water was removed from the water bath, and the polymer powder, weighed in advance, was gradually added. The addition was made in small portions, stirring continuously with a mechanical stirrer, to ensure complete dissolution and homogenization of the powder in the hot water. As a result of this process, a translucent, whitish solution was obtained. Subsequently, the cold water kept in the refrigerator was gradually added, stirring with a glass rod. When the hot solution is combined with the cold water, gel formation occurs. The gel obtained was then left to rest to eliminate air bubbles formed during the preparation process.

### 3.3. Preparation of Collagen–MC Hydrogels with Indomethacin

The 1% collagen gel was combined with 3% methylcellulose gel in varying proportions, and the active substance (indomethacin) was added in different concentrations. All hydrogels obtained were cross-linked using the same concentration of glutaraldehyde (0.05%). The resulting hydrogels were subjected to lyophilization to obtain the corresponding sponge-like matrices. The hydrogels and corresponding matrices were coded in the same way, and their composition is presented in Table 2. Percentages of collagen gel, MC gel, indomethacin and glutaraldehyde are reported to 100 g gel.

### 3.4. Preparation of Sponge-like Matrices by Lyophilization

**Figure 14:**
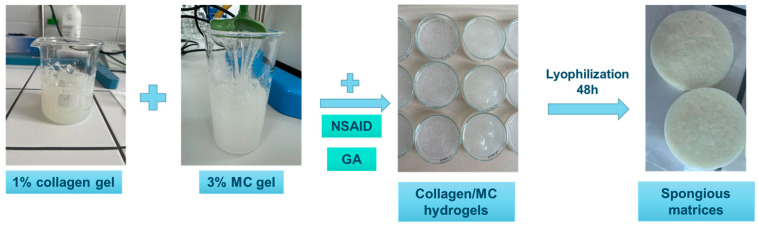
Preparation of CMI1–CMI6 sponge-like matrices.

The resulting hydrogels were poured into Petri dishes with a diameter of 5.2 cm and subsequently subjected to the lyophilization process using a program developed by the Collagen Department, Division of Leather and Footwear Research Institute, INCDTP, Bucharest, on the Delta LSC 2-24 equipment (Martin Christ, Germany). The lyophilization program lasted 48 h and started with a freezing temperature of −40 °C [110]. Following this process, six sponge-like matrices (coded CMI1–CMI6) were obtained, differentiated according to composition. The preparation process of the collagen/MC hydrogels, and their corresponding lyophilized forms, is schematically illustrated in Figure 14.

The matrices obtained by lyophilization of collagen/methylcellulose-based hydrogels were subjected to complex analyses, including spectral characterization (FT-IR analysis), morphological evaluation (scanning electron microscopy, absorption capacity), goniometric assessment (contact angle determination), biological testing (in vitro enzymatic degradation), biopharmaceutical evaluation (in vitro release kinetics of indomethacin), and pharmacological assessment through in vivo testing on Wistar rats, in order to investigate their potential as dressings for the treatment of various skin lesions.

### 3.5. Fourier Transform Infrared (FT-IR) Spectroscopy

Attenuated total reflectance Fourier transform infrared (ATR-FTIR) spectroscopy was employed to characterize the CMI1–CMI6 samples, using a Jasco FT/IR-4X spectrometer (Jasco, Tokyo, Japan) equipped with an ATR PRO ONE module (Jasco, Tokyo, Japan). Infrared spectra were recorded over the 4000–500 cm^−1^ wavenumber range at a spectral resolution of 4 cm^−1^. Each reported spectrum represents the average of 64 consecutive scans.

### 3.6. Scanning Electron Microscopy (SEM)

The microtopographic structure of the sponge-like matrices obtained based on collagen and methylcellulose was investigated using the scanning electron microscopy (SEM) technique. The examination was performed using a Tabletop TM 4000 Plus microscope (Hitachi, Tokyo, Japan), without applying a conductive layer to the surface of the samples. The analyses were performed at an accelerating voltage of 15 kV, using a backscattered electron (BSE) detector.

### 3.7. Compressive Strength Testing

The compressive strength (MPa), at 50% deformation, was determined according to ISO 844:2007, *Rigid Cellular Plastics—Determination of Compression Properties* [111], at room temperature, with 0.5 mm/min, using an Instron 3382 Universal Testing Machine and three rectangular specimens of 10 × 10 × 6 mm for each sample.

### 3.8. Contact Angle Measurements

The determination of the wetting properties of the sponge-like matrices was performed by measuring the contact angle, using the CAM 101 system (KSV Scientific Instrument, Espoo, Finland). To investigate the hydrophilic character of the samples, the contact angle method was applied, suitable for analyzing materials with a porous structure, as it allows the evaluation of the interaction between the liquid and the matrix surface. Briefly, a drop of phosphate buffer (pH = 7.4) was brought onto the surface of the matrices using a Hamilton microsyringe and the shape of the drop in contact with the sample was recorded using the device’s digital camera. The first frame was used in the calculation of the contact angle value. The experiment was performed in triplicate, and the results represent the mean contact angle value.

### 3.9. Evaluation of the Absorption Capacity

The determination of the absorption capacity of the sponge-like matrices was carried out by a gravimetric method, consisting of comparing their weight before and after immersion in phosphate buffer (pH = 7.4). Samples of identical size and shape were cut from each matrix, then weighed in a dry state (mass denoted W_0_), ensuring a similar initial weight between the samples. These were subsequently immersed in 3 mL of phosphate buffer with pH 7.4. At predetermined time intervals (0.17, 0.34, 0.5, 1, 2, 3, 4, 5, 6, 7, 24, 48 and 72 h), the fragments were removed from the medium and reweighed (mass denoted W_t_). Based on the values obtained, the absorption capacity was calculated according to Equation (1). The experiment was performed in triplicate, and the reported results represent the average of the measured values.

(1) $$\mathrm{Absorption capacity}\;(g/g)=\frac{W_{t}\;-\;W_{0}}{W_{0}}$$

### 3.10. In Vitro Enzymatic Degradation

The biological evaluation of the sponge-like matrices was performed by testing the enzymatic degradation in vitro, using collagenase—a specific enzyme involved in the degradation of collagen and one of the main proteolytic enzymes present in the wound environment and involved in extracellular matrix remodeling during wound healing [112]. For this purpose, a collagenase solution (1 µg/mL) was prepared using phosphate buffer with pH 7.4 as the dissolution medium. For each sample, fragments of comparable size and mass were cut, which were initially placed in 3 mL of phosphate buffer (pH 7.4) and maintained for 24 h to reach the equilibrium state. After this stage, the hydrated matrices were weighed, and the mass obtained was noted as W_0_ (initial mass). Subsequently, the phosphate buffer was removed, and 3 mL of collagenase solution was added to each sample, followed by incubation at 37 °C. At predetermined time points (0.25, 0.5, 1, 2, 3, 4, 5, 6, 24, 48 and 72 h), the fragments were extracted and reweighed (mass denoted W_t_). The degree of enzymatic degradation was evaluated by calculating the weight loss suffered by the samples in contact with collagenase solution, according to Equation (2).

(2) $$\mathrm{Weight loss}\;(\%)=\frac{W_{0}\;-\;W_{t}}{W_{0}}\cdot 100$$

Each analysis was performed in triplicate, and the results were expressed as the average of the values obtained.

### 3.11. In Vitro Release Kinetics of Indomethacin from the Sponge-like Matrices

The in vitro release of indomethacin from the sponge-like matrices was monitored using a “transdermal sandwich” experimental system, connected to a dissolution apparatus (Essa Dissolver, Milan, Italy), as previously described [89]. Briefly, the stages of the experiment were as follows: (i) weighing the test samples and mounting the experimental device; (ii) preparation of the release medium–phosphate buffer with pH 7.4; (iii) introduction of the medium into the test vessels and bringing it to working temperature; (iv) placement of the “sandwich”-type device in the medium, once the temperature of 37 °C is reached; (v) extracting of samples (5 mL) at predetermined time points, with immediate replacement of the extracted volume with pre-warmed buffer at the same temperature; (vi) quantitative determination of indomethacin released in a predetermined time interval, performed by a spectrophotometric method, using a calibration curve made in phosphate buffer (pH 7.4); the absorption spectrum indicated a maximum absorbance value at a wavelength of 280 nm, which was used as a reference for all subsequent analyses. All experiments were carried out in triplicate. The kinetic data were fitted according to the Power law model (Equation (3)) and its particular cases, namely the Higuchi and zero-order models:

(3) $$\frac{m_{t}}{m_{\infty }}=k\cdot t^{n}$$

where m_t_/m_∞_ represents the fraction of the drug released at time t, k the rate constant of the release process (1/min^n^), n the release exponent (dimensionless), and m_t_ and m_∞_ the amounts of the drug released from the biopolymeric systems at time t and at equilibrium, respectively; in the case of the zero-order model, the value of the release exponent is equal to 1, whereas for the Higuchi model it is 0.5 [51].

Regarding drug loading, indomethacin was incorporated directly into the collagen–MC mixed gels prior to lyophilization. As the fabrication process did not include washing or solvent exchange steps that could lead to drug removal, the theoretical drug amount initially added during preparation was assumed to correspond to the incorporated dose. Therefore, a separate determination of drug loading efficiency was not performed. However, minor drug losses during processing cannot be entirely excluded and should be considered a limitation of the present study.

### 3.12. Evaluation of Wound Healing in Experimental Animal Models

The in vivo experiment was conducted on 48 male Wistar rats (180 ± 20 g) at the “Carol Davila” University of Medicine and Pharmacy, Bucharest, Romania. The study was conducted in accordance with the Directive 2010/63/EU on the protection of animals used for scientific purposes and Romanian Law No. 43/2014 and were approved by the Scientific Research Ethics Committee of the “Carol Davila” University of Medicine and Pharmacy, Bucharest (Approval No. 13618, 24 May 2024).

The animals were housed under standard laboratory conditions, receiving food twice daily and water ad libitum. To reduce potential selection bias, animals of similar age and body weight were assigned to experimental groups using a simple randomization approach. The study included multiple groups (n = 6 per group), corresponding to the tested formulations (CMI1–CMI6), alongside comparator groups treated with a collagen matrix and sterile gauze.

Prior to injury induction, hair was removed from the dorsal area of each rat under ether anesthesia. Experimental burns (10 mm diameter) were produced using a metallic accessory, previously heated in a boiling physiological saline bath, maintained at 98 ± 1 °C. The heated device was applied to the skin surface for 15 s to induce uniform deep burn wounds. The reproducibility of the injury was ensured by maintaining constant device geometry, temperature, and exposure duration.

The burns were aseptically treated and the matrices were applied, fixed in place with silk plaster. Wound size was determined using a digital caliper based on image analysis. All measurements were conducted using a standardized protocol across the entire study to maintain consistency. To enhance measurement reliability, all assessments were conducted according to a standardized protocol, and the investigator responsible for data collection was blinded to group allocation. Wound progression was documented photographically at regular intervals (every 2–3 days) over a period of 15 days. Images were acquired under standardized conditions, including consistent distance, lighting, and camera parameters.

Healing progression was quantified by calculating the percentage reduction in wound area relative to the initial measurement (Equation (4)).

(4) $$\mathrm{Healing process}\;(\%)=\frac{W_{i}\;-\;W_{t}}{W_{i}}\cdot 100$$

where W is the wound size, calculated as the average of the longest and shortest dimensions of the burn lesion; W_i_ is the initial wound size; and W_t_ is the wound size measured at different time points. Wounds were considered fully healed when the scab formed at the injury site had completely detached [89,99].

### 3.13. Statistical Analysis

Statistical analysis was conducted using GraphPad Prism version 10.3.1 and Microsoft Excel (Microsoft 365 MSO). Data are presented as mean ± standard deviation (SD), with n = 3 for physicochemical analyses and n = 6 for in vivo experiments. The Kolmogorov–Smirnov test was used to assess normality. For the in vitro absorption capacity evaluation, statistical comparisons among formulations were performed using two-way ANOVA followed by Tukey’s multiple comparisons test, with a significance level of *p* < 0.05. For the in vivo animal studies, group differences were evaluated using one-way or two-way ANOVA followed by Dunnett’s multiple comparison test, with a *p*-value of <0.05 considered statistically significant.

## 4. Conclusions

This work focused on the development and characterization of sponge-like matrices composed of collagen and methylcellulose, incorporating indomethacin as a model anti-inflammatory drug for wound healing applications. Overall, the developed biopolymeric systems presented suitable characteristics for use as wound dressings, with promising applications in the management of inflammatory skin lesions, strengthening the direction of future research in the field of biomaterials for topical administration. While collagen-only systems showed increased hydrophilicity and absorption capacity, CMI2 and CMI5 formulations (25% methylcellulose/75% collagen) appeared to exhibit a more favorable balance between controlled fluid uptake, sufficient structural stability, and effective drug release. The incorporation of 25% methylcellulose led to a slight reduction in hydrophilicity compared to collagen-only systems, while maintaining an overall hydrophilic character and adequate fluid absorption capacity. At the same time, the incorporation of MC resulted in a faster degradation rate compared to collagen-only systems. In this context, such a controlled degradation profile may be considered beneficial for wound healing applications, as an ideal dressing should exhibit a degradation rate compatible with the tissue regeneration process. Notably, this composition also promoted a higher drug release, which may be advantageous for achieving a more effective initial anti-inflammatory response. These properties were also reflected in the in vivo studies, where both formulations demonstrated enhanced wound healing performance. Notably, the incorporation of methylcellulose offers an additional means of tuning the system, allowing its properties to be modulated by adjusting the polymer ratio depending on the intended application. However, successful clinical translation will require further investigations into scale-up manufacturing, sterilization procedures, and comprehensive biocompatibility assessment, together with future clinical validation in relevant human wound models.

## Figures and Tables

**Table 1:** Parameters of the applied kinetic models and the cumulative percentage of indomethacin released.

Sponge	Zero-Order Model			Higuchi Model			Power Law Model			Kinetic Constant, k (1/min^n^)	Release Exponent, n	IND Released (%) 24 h
	R	Adj R^2^	AIC_c_	R	Adj R^2^	AIC_c_	R	Adj R^2^	AIC_c_			
CMI1	0.7580	0.5392	−37.70	0.9263	0.8462	−53.06	0.9663	0.9283	−63.75	0.104	0.32	89.62
CMI2	0.8229	0.6503	−38.76	0.9543	0.9033	−56.75	0.9645	0.9245	−60.23	0.057	0.41	96.09
CMI3	0.7620	0.5457	−39.69	0.9274	0.8485	−55.06	0.9611	0.9175	−63.57	0.084	0.34	81.78
CMI4	0.7603	0.5430	−40.58	0.9262	0.8460	−55.82	0.9601	0.9152	−64.17	0.082	0.34	78.61
CMI5	0.8165	0.6389	−41.37	0.9503	0.8951	−58.68	0.9604	0.9159	−61.77	0.050	0.41	84.29
CMI6	0.8263	0.6565	−46.54	0.9548	0.9045	−64.46	0.9658	0.9272	−68.26	0.046	0.40	74.34

**Table 2:** Composition of hydrogels and corresponding sponge-like matrices.

Code	Collagen Gel * (%)	Methylcellulose * (%)	Indomethacin (%)	Glutaraldehyde (%)
CMI1	100	0	0.1	0.05
CMI2	75	25	0.1	0.05
CMI3	50	50	0.1	0.05
CMI4	100	0	0.2	0.05
CMI5	75	25	0.2	0.05
CMI6	50	50	0.2	0.05

* 1% collagen gel, 3% methylcellulose gel.

## Data Availability

The original contributions presented in this study are included in the article. Further inquiries can be directed to the corresponding authors.
